# Effect of phototherapy on cardiac function in full term neonates with unconjugated hyperbilirubinemia

**DOI:** 10.1038/s41390-025-04071-4

**Published:** 2025-05-08

**Authors:** Doaa El Amrousy, Rowan Adel, Dalia Ayoub, Mohamed Rowisha

**Affiliations:** Pediatric Department, Tanta University Hospital, Tanta, Egypt

## Abstract

**Background:**

Phototherapy of jaundiced neonates has several cardiovascular effects. We investigated the effect of phototherapy on cardiac function in jaundiced full-term neonates using two-dimensional speckle tracking echocardiography (2D-STE).

**Methods:**

This cohort study was conducted on 40 full term neonates with unconjugated hyperbilirubinemia who needed phototherapy as the patient group and 40 healthy neonates of matched gestational age, age, and sex served as the control group. Conventional echocardiography, tissue Doppler, and 2D-STE were performed before and 24 h after phototherapy. Serum troponin T, serum bilirubin levels, systolic and diastolic blood pressure, and heart rate (HR) were measured also before and after phototherapy.

**Results:**

Systolic and diastolic blood pressure significantly decreased in the patient group after phototherapy compared to their levels before phototherapy. On the contrary, HR significantly increased after phototherapy in the patient group. However, serum cardiac troponin showed non-significant change after phototherapy. After phototherapy, all conventional and tissue Doppler parameters showed non-significant change. While 2D- left ventricular global longtudinal strain (2D-LVGLS) obtained by STE significantly decreased after phototherapy. There was a significant negative correlation between 2D-LV GLS and both duration and number of phototherapy units and both are good predictors for 2D-LVGLS.

**Conclusion:**

Phototherapy may cause myocardial dysfunction that is correlated with the duration and number of phototherapy units.

**Impact:**

- Phototherapy of jaundiced neonates has many cardiovascular effects such as decrease in blood pressure and stroke volume. However, the effect of phototherapy on cardiac function in neonates has not been fully investigated yet.
- We investigated, for the first time, the effect of phototherapy on cardiac function in jaundiced full term neonates using two-dimensional speckle tracking echocardiography (2D-STE).
- Phototherapy may cause myocardial dysfunction that is correlated with the duration and number of phototherapy unit. Cardiac shield may be needed during phototherapy.

## Introduction

Neonatal hyperbilirubinemia is a common medical condition in newborn infants.^1^ Phototherapy is the standard of care for unconjugated hyperbilirubinemia when treatment is necessary.^2^ Phototherapy is considered effective and safe treatment; however, several adverse effects have been reported. These adverse effects include deoxyribonucleic acid damage as well as other impacts on the newborn’s organs such as retina, skin, testis, and cardiovascular system.^3^

Phototherapy has been reported to alter cardiovascular hemodynamics by increasing sympathetic activity, lowering cardiac output, and increasing peripheral blood resistance, which may be concerning in premature or ill newborns.^4^

Echocardiography is essential for evaluating overall myocardial function. Echocardiography offers several advantages; it is non-invasive, does not involve radiation, and is simple and easy to operate.^5^ Two-dimensional speckle tracking echocardiography (2D-STE) is a developed method to evaluate local and global myocardial function.^6^ It enables cardiologists to track myocardial tissue movement and to detect early myocardial dysfunction compared to conventional echocardiography before clinical signs develop.^7–9^

We conducted the current study to evaluate the cardiac function and hemodynamic changes in full-term neonates with hyperbilirubinemia that needs phototherapy.

## Subjects and methods

This prospective cohort study was conducted at neonatal intensive care unit of Tanta University Hospitals. The local ethical committee of faculty of medicine, Tanta University approved the study (No.35631/8/22). All parents of included neonates signed a written consent before the enrollment. Forty full term neonates with unconjugated hyperbilirubinemia who needed phototherapy were included in the study as the patient group during the period from December 2022 to October 2023. Forty healthy neonates of matched gestational age, age, and sex served as the control group.

Inclusion criteria: full term neonates with pathological unconjugated hyperbilirubinemia who needed phototherapy according to the American society of pediatrics guidelines.^10^

We used phototherapy devices (MediLED, Germany) which used blue light-emitting diodes (LED) light source for the treatment of neonates with unconjugated hyperbilirubinemia. This device, LED, can deliver narrow-spectrum light at any given wavelength.

Exclusion criteria: neonates with gestational age less than 37 weeks, small for gestational age, major congenital abnormalities, congenital heart diseases, respiratory and circulatory problems, neonatal sepsis, neonates with dehydration, acid-base disorder, or electrolyte imbalance, inborn errors of metabolism, bilirubin encephalopathy, intrauterine growth restriction, and perinatal asphyxia were excluded.

All neonates were subjected to perinatal history including maternal illness, mode of delivery, Apgar score, history of cyanosis or convulsions. Clinical examination with special emphasis on vital signs, anthropometric measures, presence of cephalhematoma, and neurological examination were performed for all study cases. Routine laboratory investigations included complete blood count, blood group and Rh typing of the neonate and mother, liver and kidney function, serum bilirubin levels (total, direct, and indirect) were measured. Serum calcium and cardiac troponin I levels were also evaluated before and 24 h after phototherapy.

## Echocardiography

Echocardiography was done by one operator for completely relaxed neonates either after feeding-up or using pacifier. The echocardiographic study was performed by using a GE Vivid 7 (Healthcare, Horten, Norway) with S7 and 4 V matrix real-time three-dimensional probes according to the guidelines of the American Society of Echocardiography.^11^

M-mode echocardiography and two dimensional echocardiography were used to measure the left ventricular end-diastolic dimension (LVEDD) and left ventricular end-systolic dimension (LVESD). Left ventricular fractional shortening (LV FS) ventricle was calculated using the left ventricular dimensions. LV FS = LVEDD-LVESD/LVEDD ×100%. Modified Simpson’s method was used to calculate left ventricular end-diastolic and end-systolic volumes, as well as left ventricular ejection fraction (LV EF). The apical four-chamber view was also used to measure mitral inflow velocities, with the ‘peak flow velocities of the mitral valve measured in early diastole as (E) wave and late diastole with atrial contraction as (A) wave, and mitral E/A ratio was calculated. Velocity time integral (VTI) of the left ventricular outflow tract was measured using Doppler to calculate stroke volume (SV) and hence left ventricular output (LVO).

Tissue Doppler Imaging (TDI) was also use to assess the LV systolic and diastolic function. At the apical four-chamber view, pulse-wave sample volume was put on the left ventricular septal mitral annulus. Systolic velocity (S’), early diastolic velocity (E’), late diastolic velocity (A’), and mitral E’/A’ ratio were measured. The Tei index or left ventricular myocardial performance index (LV MPI) was calculated.^12^

### Two-dimensional speckle tracking echocardiography

We used image of the apical 4-chamber view, the apical 2-chamber view, and the apical 3-chamber view to calculate left ventricular global longitudinal strain (LV GLS). We traced the endocardial envelope accurately and ensured the region of interest contains complete ventricular wall information. Finally, we clicked the approve key to confirm the tracking and the software calculates LV GLS automatically. The mean of three cardiac cycles was taken.

Echocardiographic examination was performed for the patients and control groups before phototherapy and they were repeated only for the patients group 24 h after the end of last session of phototherapy.

### Statistical analysis

Statistical analysis was performed with the Statistical Package for the Social Sciences version 20.0 (SPSS Inc., Chicago, IL, US). Continuous data are presented as mean and standard deviation (SD) if normally distributed, or median and interquartile range if not-normally distributed. Discrete data are presented as absolute values and percentages. Continuous variables were compared between the two groups with Student’s t-test or Mann–Whitney U test when appropriate. Paired T test was used to compare continuous variables within the same group before and after phototherapy. Categorical variables were compared with chi squared test, or Fisher exact test, when appropriate. Correlations were assessed using either Pearson’s correlation test or Spearman’s rank test according to the distribution pattern of the variable. Linear regression analysis was performed to assess the possible predictors for 2D-LV GLS in the patient group. A p-value of <0.05 was considered statistically significant.

## Results

The demographic, clinical, and laboratory data of the studied groups before phototherapy were shown in Table 1. There was no significant difference between the two groups as regards postnatal age, gestational age, sex, weight, APGAR score at 5 m, systolic blood pressure (SBP), diastolic blood pressure (DBP), heart rate (HR), hemoglobin (Hb) levels, alanine transaminase (ALT), aspartate transaminase (AST), urea, creatinine, direct bilirubin, or serum cardiac troponin I. However, total and indirect bilirubin were significantly elevated in the patient group compared to the control group. Regarding the cause of jaundice in the patient group, twenty-nine neonates (72.5%) had ABO incompatibility, nine neonates (22.5%) had RH incompatibility, and two neonates (5%) had glucose 6-phosphate dehydrogenase deficiency (G6PD). The mean duration of phototherapy in our patients was 4.3 ± 1.5 days. Ten cases (25%) had single phototherapy, five cases (12.5%) had double phototherapy, 17 cases (42.5%) had triple phototherapy, and eight cases (20%) were in capsules; equivalent to four phototherapy devices. (Table 1).

**Table 1:** Neonate’s characteristics of the studied groups before phototherapy.

Parameters	Patient group before phototherapy	Control group	*P* value
Postnatal age (days) (min-max)	2.7 ± 1.4 (1–8)	3.2 ± 1.9 (1–11)	0.091
Gestational age (w) (min-max)	38 ± 0.8 (37–39)	38.3 ± 0.7 (37–40)	0.590
Sex (male: female)	20:20	15:25	0.260
Weight (kg)	3.7 ± 0.4	3.6 ± 0.4	0.255
SBP (mmHg)	95.4 ± 5.2	94.6 ± 8.2	0.604
DBP (mmHg)	53.9 ± 5.5	52.9 ± 6.4	0.455
HR (b/m)	123.3 ± 8.9	121.6 ± 7.2	0.371
APGAR score (5 m) (min-max)	8.2 ± 0.8 (7–10)	8.4 ± 0.5 (8–10)	0.182
ALT (U/L)	25.8 ± 6.8	23.8 ± 6.4	0.184
AST (U/L)	24.9 ± 6.4	23.4 ± 5.2	0.245
Hemoglobin (g/dl)	13.3 ± 0.9	14.1 ± 1.5	0.081
Urea (mg/dl)	29.1 ± 5.9	27 ± 7.7	0.187
Creatinine (mg/dl)	0.4 ± 0.2	0.4 ± 0.1	0.322
Total bilirubin (mg/dl)	17.4 ± 1.9	4.9 ± 0.8	<0.001
Direct bilirubin (mg/dl)	0.4 ± 0.1	0.3 ± 0.1	0.154
Indirect bilirubin (mg/dl)	17 ± 1.9	4.6 ± 0.8	<0.001
Serum cardiac troponin			
- Normal level	40 (100%)	40 (100%)	
- Abnormal level	0 (0%)	0 (0%)	
Causes of jaundice:			
-ABO incompatibility	29 (72.5%)	-------	
- Rh incompatibility	9 (22.5%)	-------	
- G6PD	2 (5%)	-------	
Number of phototherapy:			
- Single	10 (25%)		
- Double	5 (12.5%)		
- Triple	17 (42.5%)		
- Capsule	8 (20%)		
Duration of phototherapy (days)	4.3 ± 1.5		

*SBP* systolic blood pressure, *DBP* diastolic blood pressure, *HR* heart rate, *ALT* alanine transaminase, *AST* aspartate aminotransferase, *G6PD* glucose 6 phosphate dehydrogenase.

Table (2) showed clinical and laboratory data in the patient group before and after phototherapy. SBP, DPB, total serum bilirubin, and indirect bilirubin level significantly decreased in the patient group after phototherapy compared to their levels before phototherapy. On the contrary, HR significantly increased after phototherapy compared to its reading before phototherapy. However, serum cardiac troponin as well as direct bilirubin showed non-significant change after phototherapy compared to their levels before phototherapy.

**Table 2:** Clinical and laboratory data in the patient group before and after phototherapy.

Parameters	Patient group before phototherapy	Patient group after phototherapy	*P* value
SBP (mmHg)	95.4 ± 5.2	89.4 ± 4.7	<0.001
DBP (mmHg)	53.9 ± 5.5	47.4 ± 4.9	<0.001
HR (b/m)	123.3 ± 8.9	130.1 ± 7.9	0.02
**Serum cardiac troponin**			
- Normal levels	40 (100%)	40 (100%)	
- Abnormal levels	0 (0%)	0 (0%)	
Total bilirubin (mg/dl)	17.4 ± 1.9	6.8 ± 1.3	<0.001
Direct bilirubin (mg/dl)	0.4 ± 0.1	0.3 ± 0.1	0.067
Indirect bilirubin (mg/dl)	17 ± 1.9	6.4 ± 1.3	<0.001

*SBP* systolic blood pressure, *DBP* diastolic blood pressure, *HR* heart rate.

There was no significant difference between the patient group before phototherapy and the control group as regards LVEDD. LVESD, LVFS, LVEF, SV, LVO, mitral S’, mitral E’/A’, LV MPI, and 2D-GLS (*P* > 0.05).

After phototherapy, all conventional and tissue Doppler parameters in the form of LVEDD. LVESD, LVFS, LVEF, mitral S’, mitral E’/A’, and LV MPI showed non-significant change compared to their pre-phototherapy values and compared to the control group. While, SV and LVO significantly decreased after phototherapy compared to their pre-phototherapy values and compared to the control group. Moreover, 2D-LVGLS obtained by STE significantly decreased after phototherapy compared to its pre-phototherapy values and compared to the control group. (Table 3)

**Table 3:** Echocardiographic parameters in the patient group before and after phototherapy compared to the control group.

Parameters	Patient group before phototherapy	Patient group after phototherapy	Control group
LVEDD (mm)	18.7 ± 1.6	18.3 ± 2.1	18.4 ± 1.5
LVESD (mm)	11.2 ± 1.3	11 ± 1.5	10.71 ± 1.48
LV FS%	40.3 ± 3.9	39.9 ± 3.8	41.5 ± 5.9
LV EF %	78.3 ± 4.1	78 ± 4.2	79.89 ± 5.91
SV (ml)	4.2 ± 0.9	3.8 ± 0.8^a,b^	4 ± 0.7
LVO (L/m)	0.57 ± 0.09	0.47 ± 0.08^a,b^	0.55 ± 0.11
Mitral S’ wave	5.2 ± 0.9	5 ± 0.6	5.4 ± 1
Mitral E’/A’	1 ± 0.08	0.99 ± 0.07	1.1 ± 0.1
LV MPI	0.42 ± 0.13	0.40 ± 0.03	0.41 ± 0.04
2D-LVGLS%	−20.6 ± −1.6	−17.9 ± −1.4^a,b^	−20.7 ± 0.7

*LVEDD* left ventricular end-diastolic dimension, *LVESD* left ventricular end-systolic dimension, *LV FS* left ventricular fractional shortening, *LV EF* left ventricular ejection fraction, *SV* stroke volume, *LVO* left ventricular output, *S*’ peak annular velocity at systole, *E’/A’* early diastolic velocity (E’)/ late diastolic velocity (A’), *LV MPI* left ventricular myocardial performance index, *2D-LVGLS* two-dimensional left ventricular global longitudinal strain.

^a^indicates significant difference between pre- and post-phototherapy variables in the patient group using paired t test.

^b^indicates significant difference between post-phototherapy variables in the patient group and the control group using student T test.

There was no significant correlation between 2D-LVGLS and postnatal age, gestational age, weight, SBP, DBP, HR, total bilirubin, LVEDD, LVESD, LV FS%, LV EF%, mitral S’, mitral E’/A’, or LV MPI. There was a significant positive correlation between 2D-GLS and SV and LVO. However, there was a significant negative correlation between 2D-LVGLS and both duration and number of phototherapy. (Table 4).

**Table 4:** Correlation between 2D-GLS and different parameters in the patient group after phototherapy.

Parameters	2D-LVGLS	
	*r*	*P*
Age (days)	0.139	0.396
Gestational age (weeks)	0.308	0.053
Weight (kg)	−0.052	0.749
SBP (mmHg)	0.124	0.445
DBP (mmHg)	0.056	0.732
Total bilirubin (mg/dl)	−0.059	0.717
Hemoglobin (g/dl)	0.010	0.953
LVEDD (mm)	0.128	0.432
LVESD (mm)	0.036	0.825
LV FS%	0.146	0.369
LV EF %	0.002	0.990
SV (ml)	0.810	0.01
LVO (L/m)	0.701	0.03
Mitral S’ wave	0.119	0.464
Mitral E’/A’	−0.102	0.531
LV MPI	−0.256	0.111
Duration of phototherapy	−0.460	0.003
Number of phototherapy	−0.361	0.02

*SBP* systolic blood pressure, *DBP* diastolic blood pressure, *LVEDD* left ventricular end-diastolic dimension, *LVESD* left ventricular end-systolic dimension, *LV FS* left ventricular fractional shortening, *LV EF* left ventricular ejection fraction, *SV* stroke volume, *LVO* left ventricular output, *S’* peak annular velocity at systole, *E’/A’* early diastolic velocity (E’)/ late diastolic velocity (A’), *LV MPI* left ventricular myocardial performance index, *2D-LVGLS* two-dimensional left ventricular global longitudinal strain.

When linear regression was performed, DBP and duration and number of phototherapy were the only predictors of 2D-LVGLS (standardized *β* = 0.437, 95% CI: 0.015–0.230; *P* = 0.027, standardized *β* = −0.367, 95% CI: −0.646 to −0.030; *P* = 0.03, and standardized *β* = −0.424, 95% CI: −1.028 to −0.055; *P* = 0.03 respectively). (Table 5).

**Table 5:** Linear regression analysis for the predictors of 2D-LVGLS in the patient group.

Independent variables	Standardized Coefficients (Beta)	*P* value	95% CI	
Age (y)	0.076	0.632	−0.420	0.679
Weight (kg)	−0.023	0.884	−1.194	1.035
HR (b/m)	0.238	0.119	−0.011	0.094
SBP (mmHg)	0.177	0.365	−0.062	0.167
DBP (mmHg)	0.437	0.027	0.015	0.230
Total bilirubin (mg/dl)	0.200	0.480	−0.287	0.594
Hemoglobin (g/dl)	0.023	0.928	−0.758	0.829
LV S	0.101	0.750	−149.445	204.695
LV MPI	−0.034	0.823	−3.647	2.928
Duration of phototherapy (days)	−0.367	0.033	−0.646	−0.030
Number of phototherapy	−0.424	0.031	−1.028	−0.055

*CI* confidence interval, *HR* heart rate, *SBP* systolic blood pressure, *DBP* diastolic blood pressure, *LV S* left ventricular S wave, *LV MPI* left ventricular myocardial performance index.

## Discussion

The result of our study showed that phototherapy might cause myocardial dysfunction that was correlated to the duration and number of phototherapy. For the best of our knowledge, the effect of phototherapy on the hemodynamics and cardiac function has not been studied well in neonates.^13,14^

Some studies suggested that there is increase in the peripheral circulation, decrease in SBP and increase in respiratory and heart rate while giving phototherapy. Therefore, it appeared that there is some effect of phototherapy on cardiorespiratory activity.^15,16^ The aims of our study were to assess the hemodynamic changes and early myocardial dysfunction (if any) in full-term neonates with hyperbilirubinemia who needed phototherapy.

In our study, there was a significant decrease in both systolic and DBP but a significant increase of HR after phototherapy compared to their levels before phototherapy. This was in line with that of Zhou et al.^17^ who reported increase in HR and decrease in the SBP during phototherapy.

Hemodynamic homeostasis is normally maintained by two important vasoactive substances (endothelin [ET] and nitric oxide [NO]). When the NO:ET ratio increases, it causes vasodilation and low blood pressure; when arterial blood pressure decreases, the body adjusts through the baroreceptor reflex negative feedback loop, that weakens the reflex, so increasing the heart rate. Liu et al.^18^ documented that phototherapy may affect the levels of endothelin (ET) and NO in the blood, thus altering the hemodynamics of neonates.

The decrease in systemic blood pressure is caused by the thermal effect exerted by phototherapy leading to cutaneous vasodilatation by axon reflexes and hormonal mechanism (nitric oxide). Moreover, nitric oxide has a direct effect on the cardiac pacemaker increasing the heart rate. On the other hand, decrease systemic blood pressure together with reducing the venous return during phototherapy lead to stimulation of the baroreceptors at the right atrium resulting in increasing heart rate.^13^ In line with our results, Takasande et al.^19^ reported that HR significantly increased in the neonates after phototherapy compared to their mean levels before phototherapy.

Our results reported that SV and LVO significantly decreased after phototherapy in the patient group. These circulatory changes in the form of significant decrease in SV and LVO is likely due to the peripheral vasodilatation; occurred as phototherapy effect; leading to decrease in venous return and stoke volume as well, resulting in decrease in systemic blood pressure and systemic blood flow including coronary blood flow that may aggravate cardiac injury.^13^ Other researchers reported that the decrease of SV might be due to the newborn’s reduced activity during phototherapy. On the other hand, other researchers documented that the left ventricular outflow decreased after the initiation of phototherapy and it returned to the pre-phototherapy levels within 12  of phototherapy.^19^

Conventional echocardiographic parameters in our study failed to reveal any significant difference before and after receiving phototherapy in the patient group. In harmony with our results, other researchers reported that there were no significant differences regarding LVEF, LVFS, LVESD, LVEDD between patients and control groups.^20,21^

Moreover, the current study revealed that different tissue Doppler parameters failed to detect any significant difference in the patients group before and after receiving phototherapy. This was in agreement with the result of Takasande et al.^19^ who studied the effect of phototherapy on cardiac function and they reported that tissue Doppler parameters were comparable in neonates before and after receiving phototherapy, concluding that phototherapy had no adverse effects on left ventricular systolic function in neonates with hyperbilirubinemia.

Our study assessed cardiac troponin I in the patients group before and after receiving phototherapy showed non-significant change denoting no overt myocardial injury can be detected. Serum cardiac troponin I showed no increase at the neonates enrolled in this study as myocardial injury may be subtle that couldn’t be detected by cardiac troponins.

Regarding 2D-LVGLS obtained by STE, our study revealed that there was a significant decrease of 2D-LVGLS in the patient group after phototherapy compared to its measure before phototherapy. Chen et al.^5^ reported that conventional LVEF becomes abnormal only when cardiomyocytes are severely damaged or the disease is relatively advanced and the myocardial injury is difficult to reverse at that time. LVEF in the patient group was in the normal range and wasn’t statistically different from that of the control group indicating that the overall LV systolic function wasn’t affected by phototherapy, therefore the LVEF measurement couldn’t detect the difference in LV systolic function between the two groups unless that is severely affected, unlike the 2D-LVGLS that could detect the subclinical changes in LV myocardial function in several diseases in pediatrics.^7–9^

2D-LVGLS being an angle independent technique in evaluation of myocardial performance makes it superior in detecting the subclinical changes in myocardial function prior to TDI and conventional echocardiography. Correlating 2D-GLS in the patients group after receiving phototherapy to different parameters, we found that 2D-GLS had a significant negative correlation with duration and number of phototherapy but a positive significant correlation with SV and LVO. Moreover, regression analysis revealed that duration and number of phototherapy are the only predictors for 2D-LVGLS in neonates with jaundice who received phototherapy.

Interestingly, our study reported that phototherapy could affect cardiac function; however, the exact mechanism is not well known. However, decreasing mean arterial blood pressure and COP may play a role but it is not the main role in cardiac affection. Further research should be performed to explore the exact mechanism of cardiac dysfunction after phototherapy and whether a cardiac shield is needed or not during phototherapy. In addition, we have to investigate the proper suitable cardiac shield needed as one previous study already tried a cardiac shield made from food grade aluminum foil during phototherapy in neonates with hemodynamically significant PDA.^22^

Limitations of the study: small sample size and short duration of follow up. Echocardiographic examination follow-up for healthy neonates was not performed. Repeated cardiac examinations during phototherapy were not performed, so the study could not reveal the safe duration of phototherapy before cardiac dysfunction appeared.

## Conclusions

Phototherapy may cause myocardial dysfunction that is correlated to the duration and number of phototherapy units. Cardiac shield may be needed during phototherapy.

## Data Availability

The data support the findings of this study are available on request from the corresponding author.

## References

[1] Kemper, A. R. et al. Clinical practice guideline revision: management of hyperbilirubinemia in the newborn infant 35 or more weeks of gestation. *Pediatrics* **150**, e2022058859 (2022). 35927462 10.1542/peds.2022-058859

[2] Faulhaber, F. R. S., Procianoy, R. S. & Silveira, R. C. Side effects of phototherapy on neonates. *Am. J. Perinatol.* **36**, 252–257 (2019). 30081405 10.1055/s-0038-1667379

[3] Tatli, M. M., Minnet, C., Kocyigit, A. & Karadag, A. Phototherapy increases DNA damage in lymphocytes of hyperbilirubinemic neonates. *Mutat. Res.* **654**, 93–95 (2008). 18534897 10.1016/j.mrgentox.2007.06.013

[4] Javorka, K. et al. Cardiovascular changes during phototherapy in newborns. *Physiol. Res.* **71**, S179–S186 (2022). 36647906 10.33549/physiolres.935002 PMC9906667

[5] Chen, Z. et al. Early changes in left ventricular myocardial function by 2D speckle tracking layer-specific technique in neonates with hyperbilirubinemia. *Quant. Imaging Med. Surg.* **12**, 796–809 (2022). 34993119 10.21037/qims-21-197 PMC8666729

[6] Yu, H. et al. Associations between neonatal serum bilirubin and childhood hypertension. *PLoS ONE* **14**, e0219942 (2029). 10.1371/journal.pone.0219942 PMC6638957 31318924

[7] El Razaky, O., El Amrousy, D., Elrifaey, S., Elgendy, M. & Ibrahim, W. Three-dimensional speckle tracking echocardiography: Is it the magic wand in the diagnosis of subclinical myocardial dysfunction in children with type 1 diabetes mellitus?. *Echocardiography* **35**, 1657–1663 (2018). 29981180 10.1111/echo.14095

[8] El Amrousy, D., El-Afify, D., Khedr, R. & Ibrahim, A. M. Omega 3 fatty acids can reduce early doxorubicin-induced cardiotoxicity in children with acute lymphoblastic leukemia. *Pediatr. Blood Cancer* **69**, e29496 (2022). 34842343 10.1002/pbc.29496

[9] El Amrousy, D. et al. Myocardial function using two dimension speckle tracking echocardiography in children with celiac disease. *Eur. J. Pediatr.* **183**, 947–954 (2024). 38060017 10.1007/s00431-023-05343-z PMC10912273

[10] American Academy of Pediatrics Subcommittee. Hyperbilirubinemia.Management of hyperbilirubinemia in the newborn infant 35 or more weeks of gestation. *Pediatrics* **114**, 297–316 (2004). 15231951 10.1542/peds.114.1.297

[11] Lopez, L. et al. Guidelines for performing a comprehensive pediatric transthoracic echocardiogram: recommendations from the american society of echocardiography. *J. ASE*10.1016/j.echo.2023.11.015 (2024). 10.1016/j.echo.2023.11.015 38309834

[12] Cui, W. & Roberson, D. A. Left ventricular Tei index in children: comparison of tissue Doppler imaging, pulsed wave Doppler, and M-mode echocardiography normal values. *J. Am. Soc. Echocardiogr.* **19**, 1438–1445 (2006). 17138026 10.1016/j.echo.2006.06.006

[13] Javorka, K., Matasova, K., Javorka, M. & Zibolen, M. Mechanisms of cardiovascular changes of phototherapy in newborns with hyperbilirubinemia. **72**, S1–S9 (2023) 10.33549/physiolres.935018 PMC10292817 37294113

[14] Uchida, Y. et al. Non-invasing monitoring of bilirubin photoisomer excretion during phototherapy. *Sci. Rep.* **12**, 11798–11823 (2022). 35821401 10.1038/s41598-022-16180-9 PMC9276810

[15] Taksande, A., Vagha, J. & Rupesh, R. Is there any effect of Phototherapy on Cardiac Function in Neonates with Hyperbilirubinemia. *Eur. J. Mol. Clin. Med.* **7**, 1967–1976 (2020).

[16] Ebbesen, F., Hansen, T. & Maisels, M. Update on phototherapy in jaundiced neonates. *Curr. Pediatr. Rev.* **13**, 176–180 (2017). 28721812 10.2174/1573396313666170718150056

[17] Zhou, S., Wu, X., Ma, A., Zhang, M. & Liu, Y. Analysis of therapeutic effect of intermittent and continuous phototherapy on neonatal hemolytic jaundice. *Exp. Ther. Med.* **17**, 4007–4012 (2019). 30988782 10.3892/etm.2019.7432 PMC6447920

[18] Liu, G. et al. Effect of phototherapy on blood endothelin and nitric oxide levels: clinical significance in preterm infants. *World J. Pediatr.* **4**, 31–35 (2008). 18402249 10.1007/s12519-008-0006-x

[19] Taksande, A., Vagha, J. & Dalal, Y. Effect of phototherapy on cardiac functions in neonates with hyperbilirubinemia. a prospective cross-sectional study. *Ann. Neonatol. J.* **3**, 88–107 (2021).

[20] Taksande, A. M. & Pujari, D. Evaluation of cardiac function in neonates with unconjugated hyperbilirubinemia. *J. Pediatr. Neonat. Individ. Med.* **11**, e110103 (2022).

[21] Karabulut, B. & Şimşek, A. Effect of neonatal hyperbilirubinemia on ventricular functions. *EJCM* **7**, 142–146 (2019).

[22] Kapoor, S., Mishra, D., Chawla, D. & Jain, S. Chest shielding in preterm neonates under phototherapy- a randomised control trial. *EJP* **180**, 767–773 (2021). 10.1007/s00431-020-03763-9 32813124

